# Legal Notes for Institutional Workers

**Published:** 1916-03-25

**Authors:** 


### LEGAL NOTES
### FOR INSTITUTIONAL WORKERS.

The question which came up for settlement in the appeal *Attorney-General* v. *Derbyshire County Council*, 32 T.L.R. 93, was as to the true construction of the Hospitals Isolation Act 1893 in reference to the control which a County Council have a right to exercise over a district committee. On the one hand it was urged that the County Council retained under the Act a control which they were entitled to exercise even to the extent of removing a committee who differed from them on matters of policy. On the other hand it was submitted that, once constituted, the District Committee were an independent body over whose action the County Council had at most only very limited powers of control. The matter, of course, turned on a construction of the Act. Section 9 provides for the making of an Order by a County Council constituting a hospital district, and directing an isolation hospital for that district to be established. Section 18 deals with the question of expenses where the hospital district consists of more than one local area and provides for contribution being made to the common fund in such proportions as the County Council by their Order constituting the district may determine. Section 20, upon the construction of which the case largely turned, is in the following terms: "A County Council may, on the application of a hospital committee, and with the assent of any local authority concerned in such alteration, alter any Order made by them for the establishment of a hospital."

What the County Council did in the case referred to was not to alter their original directions as to the constitution or establishment of the hospital or hospital district, but to alter the constitution of the committee so as to place the members of the County Council in a substantial majority, and in order to effect that result they decreed that the existing committee should cease to exist as from a certain date. In so prescribing the County Council proceeded on Section 10, which provides that where a hospital district has been constituted a committee shall be formed by the County Council who shall make regulations for the election, regulation, rotation, and for all other matters relating to the constitution of any such committee.

### The Proverbial Coach and Four.

The Divisional Judge (Sargant, J.) found that the County Council did not possess the unlimited power claimed, and with this opinion the Master of the Rolls agreed, but the majority of the Court of Appeal held that the County Council had power to alter the constitution of the committee. "The conclusion at which I have arrived," said Lord Justice Bankes, who gave the fullest judgment, "is that the whole scope and intention of the Act were to set up committees which should not be independent of the County Councils, but which should be subservient to them even to the extent, acted upon in the present instance, of wiping them out of existence." Section 20, which provides expressly for the assent of the local committees, was considered by the majority Judges to have no reference to the committee, but only to "the establishment" of the hospital. Therefore it would seem that while the original Order setting up a hospital cannot be altered without the committee's consent, a council wishing to make an alteration may effect their design by arranging for a subservient committee.

### The Surgeon's Responsibility.

We notice some discussion in American legal journals over the recent determination of a physician in a Chicago hospital, upon consent of a child's parents, to withhold from the child the benefits of an operation that would have saved its life, the reasoning leading to such a conclusion being that the child was a monstrosity in deformity and probable lack of brain power, and in its own interests and the interests of society was better dead. The severely legal view is forcibly put forward, first, that there is no distinction in law between the right of a defective child to life and one that is normal; and, second, that until the law provides such a distinction by express legislation, to assume one and act on it is criminal. The *Central Law Journal* thus concludes its editorial on the case: "We do not wish to assail the doctor's moral view; we have nothing in a law journal to say about that. We do say, however, that he seems never to have asked himself how he as arbiter of death would stand as to the law of the country where he lives." In view of the world-wide publicity which the incident received, we think the caution contained in the above-quoted words should be pointedly brought to public notice, and especially to that of hospital managers and medical staffs. The more advanced view, as taken by the Chicago surgeon, has been pithily expressed long ago in verse:—

> Thou shalt not kill, but needst not strive
> Officiously to keep alive.

### The Care of Defective Children.

The Mental Deficiency and Lunacy (Scotland) Act, 1913, enacts that it shall be the duty of the parents or guardians of children between five and sixteen years of age who are defectives to make provision for their education and their proper care and supervision; and, where they are unable to do so, it shall be the duty of the School Board to make such provision. The School Board and the Parish Council of Glasgow brought an agreed case for the opinion of the Court to determine whether the statute referred to transferred the maintenance of defective pauper children to the School Board. It was held that the term "parent" or "guardian" included Parish Councils, and therefore the duty of maintenance and education remained with Parish Councils unless they could prove inability.

